# Optimizing Tomato (*Lycopersicon esculentum*) Yield Under Salt Stress: The Physiological and Biochemical Effects of Foliar Thiourea Application

**DOI:** 10.3390/plants13233318

**Published:** 2024-11-26

**Authors:** Jawaria Abdul Majeed, Safura Bibi, Athar Mahmood, Liaqat Ali, Muhammad Ehsan Safdar, Mahmoud F. Seleiman, Zain Ul Abidin, Bushra A. Alhammad, Muhammad Ahsan Asghar

**Affiliations:** 1 Department of Botany, University of Agriculture Faisalabad, Faisalabad 38000, Pakistan; bssb.15.36@gmail.com (J.A.M.); safura.bibi@uaf.edu.pk (S.B.); abidin@gmail.com (Z.U.A.); 2 Department of Agronomy, University of Agriculture Faisalabad, Faisalabad 38000, Pakistan; 3 Cholistan Institute of Desert Studies, The Islamia University of Bahawalpur, Bahawalpur 63100, Pakistan; liaqatali@iub.edu.pk; 4 Department of Agronomy, College of Agriculture, University of Sargodha, Sargodha 40100, Pakistan; mehsan.safdar@uos.edu.pk; 5 Department of Plant Production, College of Food and Agriculture Sciences, King Saud University, P.O. Box 2460, Riyadh 11451, Saudi Arabia; mseleiman@ksu.edu.sa; 6 Biology Department, College of Science and Humanity Studies, Prince Sattam Bin Abdulaziz University, Al Kharj Box 292, Riyadh 11942, Saudi Arabia; b.alhamad@psau.edu.sa; 7 Department of Biological Resources, Agricultural Institute, Centre for Agricultural Research, ELKH, Brunzvik St., 2462 Martonvásár, Hungary

**Keywords:** thiourea, salinity, tomato, plant bioregulators, antioxidant, yield attributes

## Abstract

A pot experiment was conducted to investigate the role of thiourea exogenous application (0 mg/L and 100 mg/L) on the morphological, physiological, and yield traits of two varieties of tomato (Naqeeb and Nadir) under different salt stress treatments (0, 60, and 120 mM) in completely randomized design (CRD). The imposition of salinity by rooting medium showed that salt stress reduced plant height by 20%, fresh shoot weight by 50%, dry shoot weight by 78%, fresh root weight by 43%, dry root weight by 84%, root length by 34%, shoot length by 32%, shoot K^+^ by 47%, Ca^2+^ by 70%, chlorophyll a by 30%, chlorophyll b by 67%, and the number of seeds per berry by 53%, while shoot Na^+^ ions were increased by 90% in comparison to those grown with control treatment. However, the exogenous application of thiourea significantly enhanced dry root weight by 25% and the number of seeds per berry by 20% in comparison to untreated plants with thiourea when grown under salt stress. Salt stress resulted in a reduction in the number of berries, weight per berry, number of seeds per berry, and seed weight in both varieties, while thiourea foliar application increased these yield parameters. On the other hand, the Nadir variety surpassed Naqeeb in plant height (+13%), root length (+31%) and shoot length (+11%), fresh shoot weight (+42%) and dry shoot weight (+11%), fresh root weight (+29%), dry root weight (+25%), area of leaf (+26%), chlorophyll a (+32%), and chlorophyll b (+24%). In conclusion, the exogenous application of thiourea can be used to mitigate salt stress in tomato plants since it can improve the growth, physiological, and yield traits of this strategic crop.

## 1. Introduction

Tomato (*Lycopersicon esculentum* L.) is second only to the potato in the top 20 commodities grown worldwide, having a yield of more than 182 Mt (million tons) spread across more than 4.7 million ha [1]. It is a more valuable fruit and vegetable crop after potato because of having specific flavor, aroma, sweetness, juicy taste, and bright red color, which plays a significant role in its widespread and rapid adoption as necessary food worldwide [2,3,4]. Tomatoes contain different chemicals that are beneficial to health [5,6], but some stresses such as abiotic or biotic stress can negatively growth and productivity and consequently the quality of fruits in tomatoes [7,8]. Different types of abiotic stress such as salinity and drought can cause a significant reduction in the yield of different crops such as tomato [7,8] and potato (*Solanum tuberosum* L.) plants [9,10]. In addition to being vital for food and the economy, tomatoes are now the standard for research on how fleshy fruits develop [11,12].

Salinity can pose a severe hazard to the environment since it can inhibit plant growth and reduce agricultural output when irrigation is performed with saline water because the roots are in direct contact with high soil salt concentrations [13]. Almost 20% of agricultural land is negatively affected by the global salinity problem and the resulting reduction in crop production [14]. Fulfilling the rising global population’s increasing demand for food is a significant challenge for agricultural researchers [15]. According to estimates, Pakistan has 16.80 million acres of irrigated agricultural areas, of which 7% are ranked as strong salinity, 9% as low-salinity, 4% as moderate-salinity, and 6% as hybrid, while 72% are designated as salt-free areas [16].

The scarcity of high-quality water is becoming a significant challenge. This makes the usage of saline water increasingly necessary and warrants consideration immediately. By making crop plants more tolerant of salt, marginal areas can be made more productive [17]. A significant environmental stressor that lowers agricultural yields is salt. It disturbs 20% of irrigated land and affects food production by 30% [18]. Salt stress has an impact on a significant portion of agricultural areas. It causes osmotic or ionic imbalance by more ROS accumulation and disturbing plant anatomy [19,20,21,22]. Additionally, photosynthetic rate and antioxidant activities lead to a reduction in crop yield [21].

Several types of strategies have been used to mitigate these harmful injuries in plants. These strategies include nutrient applications, phytohormones application, modifying irrigation systems, fertilization, etc. [23,24].

Plant growth regulators have been shown to promote plant heat tolerance by controlling numerous metabolic processes in plants [25]. To eliminate hydroxyl or superoxide radicals, thiourea is crucial for lowering the combined compressive potential [26]. Thiourea is a sulfhydryl plant bioregulator (PBR) that promotes plant growth to mitigate the negative effects of climatic factors and increase agricultural yields [27,28]. Thiourea (ThioU) enhances sugar transfer and photosynthesis [29]. According to [30], thiourea-modified biochars positively impact the stabilization of toxic metals in soils, which enhances plant growth. Similarly, according to [31], thiourea foliar application enhanced salinity and water stress tolerance through physiological and biochemical improvements, which were related to wheat plant growth and production. Due to its water-soluble qualities, thiourea can be easily absorbed to reduce stress damage, which eventually boosts crop yields in stressful situations [32]. For the mitigation of salinity, ThioU foliar or some materials such as hormones can improve antioxidants, increases ROS detoxification, and activates the ascorbate–glutathione cycle, all of which have a pivotable role in reducing salt stress [27,33]. The main objectives of this experiment were to investigate the impact of ThioU foliar spray on the morphology and production of tomato (*Lycopersicon esculentum*), as well as to study the effect of thiourea in ameliorating salt stress in tomato.

## 2. Materials and Methods

A pot experiment was carried out at UAF Community College, PARS. The experiment was designed to examine the growth, physiological, biochemical, and yield response of two tomato varieties to the foliar application of thiourea under salinity stress. Tomato seeds of two varieties (Naqeeb and Nadir) were sown in pots filled with sand. Plants were cultivated in three replicates in plastic pots filled with sand, and the arrangement of the pots was completely randomized design “CRD”. About 6 kg of sand was placed in each pot. Each pot had proportions of about 25 cm in diameter and 30 cm in height. After a few days of germination, 5 seedlings were kept by thinning within every pot, and 250 mL of full-strength Hoagland’s nutrient solution was applied to each pot every week. Pots were divided into 3 sets and different concentrations of salt stress (0 mM, 60 mM, and 120 mM NaCl) and foliar spray (0 mgL^−1^ and 100 mgL^−1^ ThioU) were applied. The first set was designated as control, the second set was kept under 60 mM NaCl, and the third set was kept under 120 mM NaCl. Thiourea (0 mgL^−1^ and 100 mgL^−1^) was applied via foliar spraying using tween-20 (@ 0.1% as surfactant). After treatment application, three plants were removed from each replication, and the results were obtained regarding the morphological, physiological, biochemical, and yield parameters at vegetative (BBCH 29) and reproductive stages (BBCH 89 and 97).

### 2.1. Growth Parameters

Shoot, root length, and fresh weight were measured. Then, plants were kept in sunlight for one day and then oven-dried at 65 °C to measure dry weight. After 24 h, dry weight was measured. The number of leaves was determined from each plant replication. Plant height was recorded from the base (ground surface) to the main axis of the plant.

### 2.2. Chlorophyll Contents

By using the methods of Arnon [34] and Davis [35], chlorophyll *a* (Equation (1)), chlorophyll *b* (Equation (2)), and carotenoids (Equation (3)) were measured. For this calculation, 0.5 g of *Lycopersicon esculentum* fresh leaf was ground into pieces. The extract was prepared at a temperature of 10 °C overnight by using 5 mL of acetone solution (80%). Then, the extract was centrifuged at 14,000 rpm for 10 min. Absorbance was measured by spectrophotometry at 480 nm, 645 nm, and 663 nm, and content values of chlorophyll *a* and *b* and carotenoids were calculated by formulas given below.

(1) Chl. a (mg/g FW)=[12.7 (OD663)−2.69 (A645)]×V/1000×W]

(2) $$Chl.\;b\;\left(\mathrm{mg}/g\;FW\right)=\left[22.9\;\left(OD645-4.68\;\left(A663\right)\right]\times V/1000\times W\right)$$

(3) Carotenoids (mg/g FW)=[4.16(OD480) – 0.89(OD663)]×V/1000×W

where “*V*” is the extract volume (mL), and “*W*” is the weight (g).

### 2.3. Enzyme Extraction

#### 2.3.1. Superoxide Dismutase (SOD)

Superoxide dismutase (SOD) activity was analyzed using the methods described in the study by Giannopolitis and Ries [36]. As mentioned in the described method, a combination of phosphate buffer, H_2_O, Triton-X, L-methionine, nitroblue tetrazolium (NBT), enzyme extract, and riboflavin was mixed and placed into cuvettes. These cuvettes were then kept under a lamp for 15 min and subsequently measured at a wavelength of 560 nm.

#### 2.3.2. Catalase and Peroxidase

To standardize the samples, the leaf extract (0.5 g) was mixed with K_2_HPO_4_ buffer (50 mM). The mixture for determining the peroxidase (POD) activity was prepared by combining K_2_HPO_4_ buffer (7 pH), guaiacol (20 mM), H_2_O_2_ (40 mM), and the sample extract (0.1 mL). Absorbance values (@ 470 nm) were recorded for a duration of 20 s. To examine the catalase (CAT) activity, a mixture of phosphate buffer (pH 7.0) at a concentration of 50 mM, H_2_O_2_ at a concentration of 5.9 mM, and the sample extract (0.1 mL) was prepared. Absorbance (@ 240 nm) was measured every 20 s using the technique described by Chance and Maehly [37].

### 2.4. Digestion Method

For measuring the ionic content, Wolf’s method [38] was used. Dried roots (0.1 g) were added in H_2_SO_4_ (2.5 mL) and placed for 12 h at room temperature. After that, 1 mL of H_2_O_2_ 35% was added, and the mixture was heated until fumes were formed, resulting in a colorless mixture. After this, distilled H_2_O was added to the mixture up to 50 mL to dilute it, and then the mixture was filtered. By using a flame photometer, ions were determined from the filtrate.

### 2.5. Leaf Area

A leaf was placed on the surface of the leaf area meter to measure LA (leaf area). Then, leaves were plucked from the plant to measure the fresh weight of each leaf. Three leaves from each replication were soaked in distilled water separately in plastic-sealed bags for four hours at room temperature (23°) so that the leaf became fully turgid. After four hours, leaves were taken out of the distilled water, placed immediately on weighting balance, and leaf turgid weight was measured. The specific leaf area was measured by using Formula (4) given below, in which the leaf area is divided by the fresh weight of the leaf.

(4) $$Leaf-specific\;area=\frac{leaf\;area}{Leaf\;fresh\;weight}\;$$

The leaf area was multiplied by the number of plants in 1 square foot, and the leaf area index was measured by using Formula (5) as follows:

(5) Leaf area index=leaf area×plants in 1 square feet

### 2.6. Yield

At the stage of maturation, the number of berries of each replication was counted. Berries were taken at random from two plants from every pot, and then the seeds were removed manually from each berry and counted. Four berries from each replication were selected and weighed using a weighing balance. Seeds were separated from pulp and dried in sunlight for one day. When the moisture of the seeds evaporated, the seed weight was measured with a digital balance.

### 2.7. Statistical Analysis

The analysis of variance (ANOVA) was performed using SPSS 21.0 software (IBM Corp, Armonk, NY USA) for the data obtained regarding the effects of the exogenous applications of thiourea treatments and salinity levels on the growth, biochemical, and yield traits of two tomato varieties. The means for different treatments were compared using Tukey’s multiple-range test [39], and *p*-values ≤ 0.05 were considered significant. In addition, the standard errors (SEs) were calculated for each parameter and are presented above the columns in figures as bars.

## 3. Results

### 3.1. Growth Parameters

**Figure 1:**
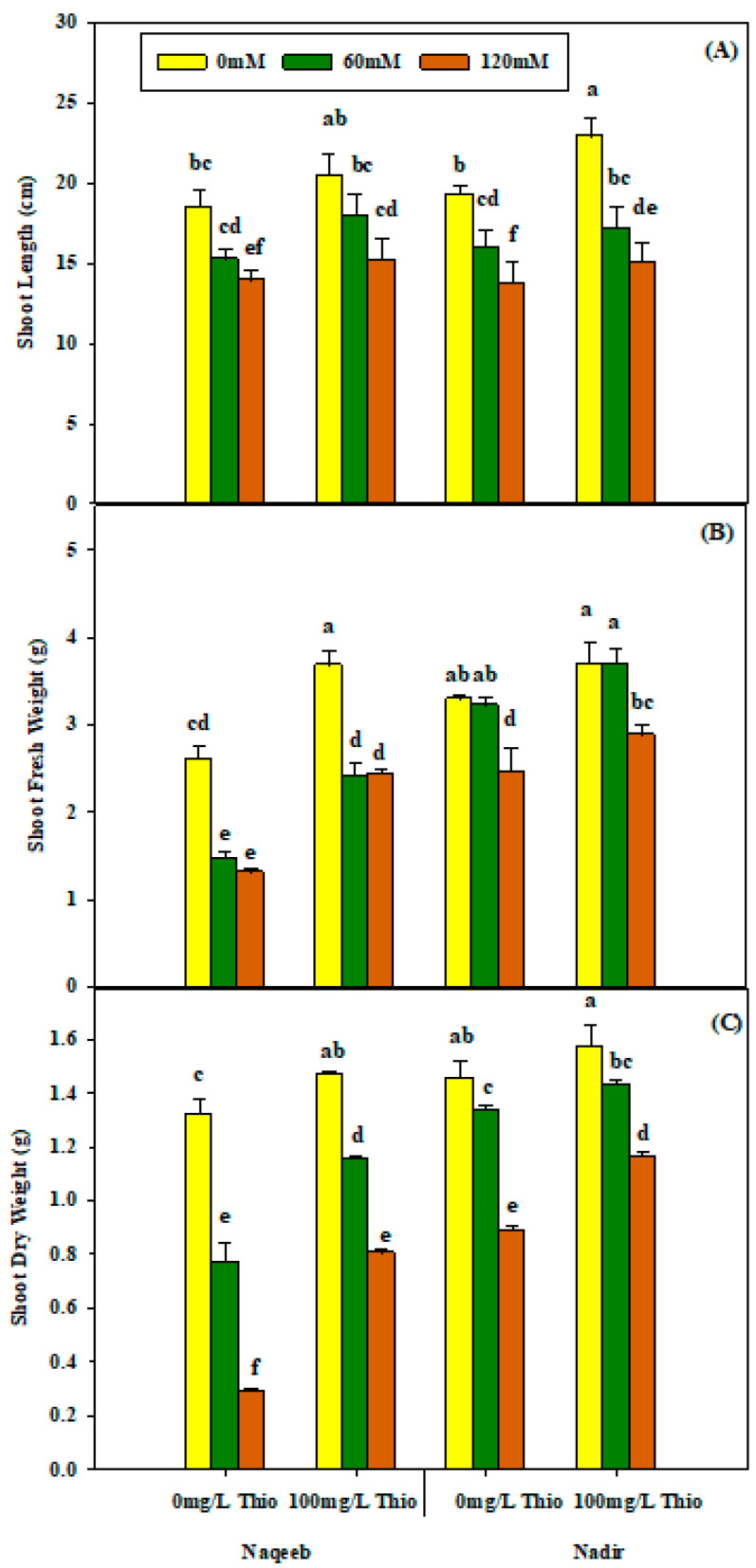
Effects of different foliar applications of thiourea on shoot length (**A**), shoot fresh weight (**B**) and shoot dry weight (**C**) of two tomato varieties grown under salinity stress. Error bars = standard error (SE), and bars with different letters indicate significant differences (*p* ≤ 0.05).

Although the salinity levels and thiourea application significantly affected the shoot length of plants as individual factors, the effect of their interaction was not significant on the shoot length of tomato plants (Table 1). Salinity treatment resulted in a non-significant decrease in shoot length. The reduction in shoot length was maximum under stress treatments (60 mM and 120 mM NaCl) as compared to nonsaline conditions. The Naqeeb variety was more affected by salinity stress as compared to the Nadir variety. The maximum shoot length was noted in the Nadir variety upon treatment with thiourea (100 mg/L) under nonsaline (0 mM NaCl) conditions. The foliar application of thiourea improved the shoot length in both varieties under different stress levels, as shown in Table 1 and Figure 1A.

A highly significant (*p* ≤ 0.05) decrease in the fresh and dry shoot weight of Nadir and Naqeeb was recorded under salinity, while non-significant results were obtained regarding salt stress, thiourea, and varieties’ interaction. It was observed that a 120 mM concentration of salt had a more significant effect on the fresh and dry weight of the shoots in Nadir and Naqeeb. The foliar application of thiourea significantly increased the fresh and dry shoot weight under nonsaline and saline conditions. However, the Nadir variety showed more improved results as compared to Naqeeb (Table 1 and Figure 1B,C).

According to the analysis of variance results, non-significant interactions were recorded for root length, root fresh weight, and root dry weight under salt stress conditions and with thiourea application (Table 1). The results showed that salinity (60 mM and 120 mM NaCl) reduced the RL, RFW, and RDW of both varieties, which were enhanced by thiourea application.

**Figure 2:**
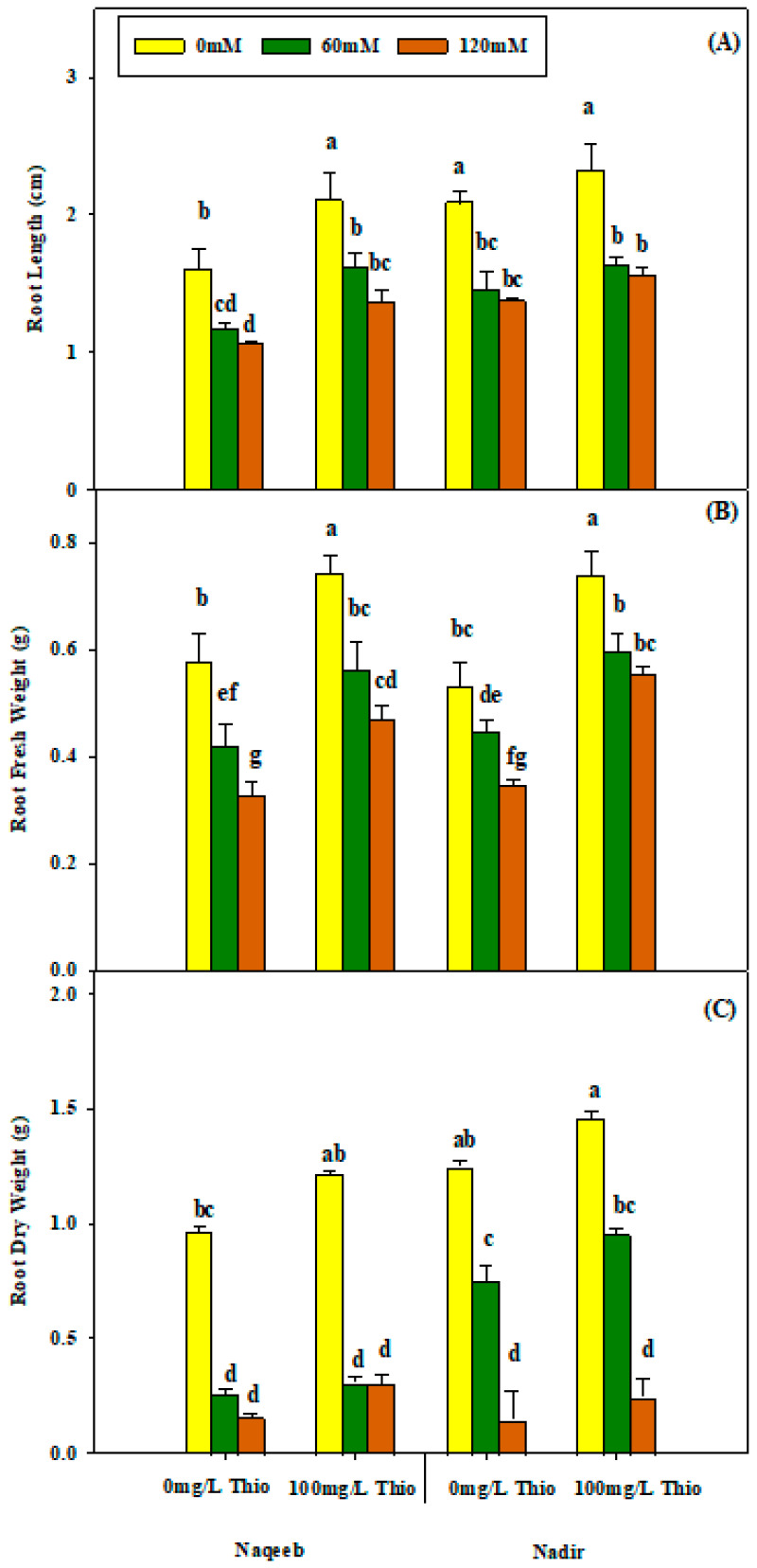
Effects of different foliar applications of thiourea on root length (**A**), root fresh weight (**B**), and root dry weight (**C**) of two tomato varieties grown under salinity stress. Error bars = standard error (SE), and bars with different letters indicate significant differences (*p* ≤ 0.05).

**Figure 3:**
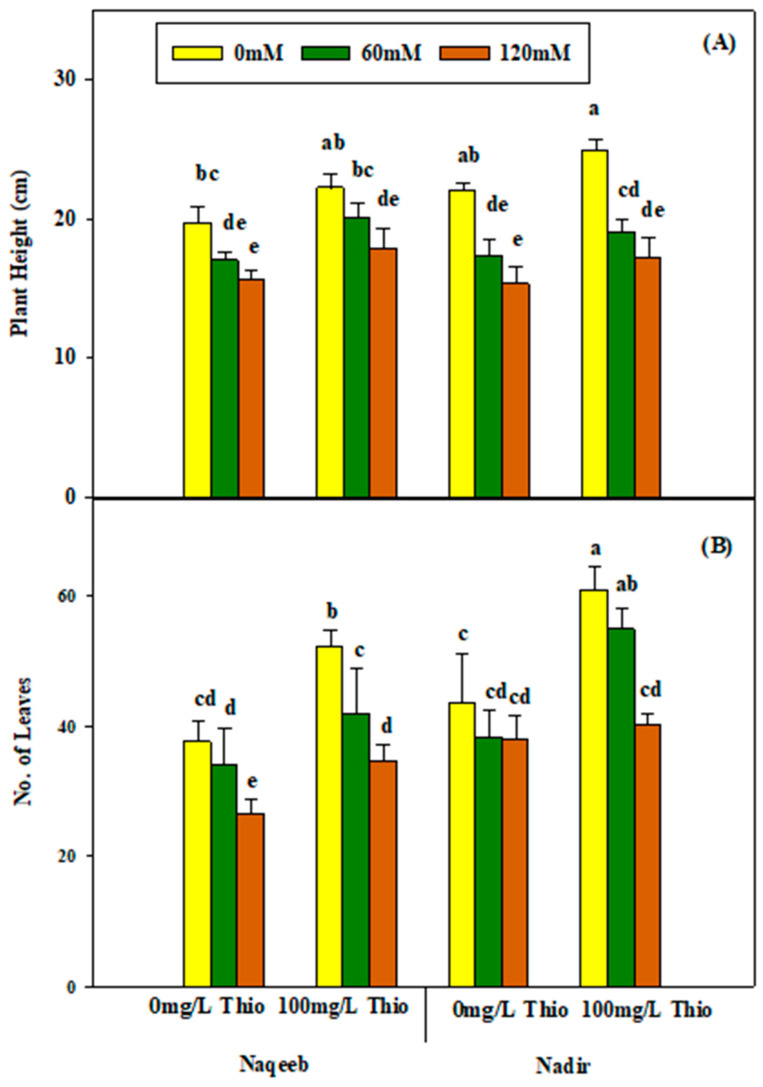
Effects of different foliar applications of thiourea on plant height (**A**) and number of leaves (**B**) of two tomato varieties grown under salinity stress. Error bars = standard error (SE), and bars with different letters indicate significant differences (*p* ≤ 0.05).

The foliar application of thiourea (100 mg/L) enhanced the RL, RFW, and RDW of both varieties in salt-stressed and nonsaline plants. However, the varieties showed non-significant differences as more improvement was noted in the RL, RFW, and RDW of the Nadir variety as compared to Naqeeb under salinity and nonsaline conditions (Table 2 and Figure 2A–C). Similarly, the plant height and number of leaves also yielded non-significant results regarding salinity and thiourea application. The plant height and number of leaves were reduced under salinity conditions (60 mM and 120 mM), and the maximum reduction was noted at the 120 mM salt level in both Naqeeb and Nadir. The foliar application of thiourea (100 mg/L) increased the total plant height and number of leaves non-significantly in both varieties under salt stress (120 mM NaCl), but this was observed only in the Nadir variety under nonsaline conditions (Table 2 and Figure 3A,B).

### 3.2. Physiological Parameters (Photosynthetic Pigments)

**Figure 4:**
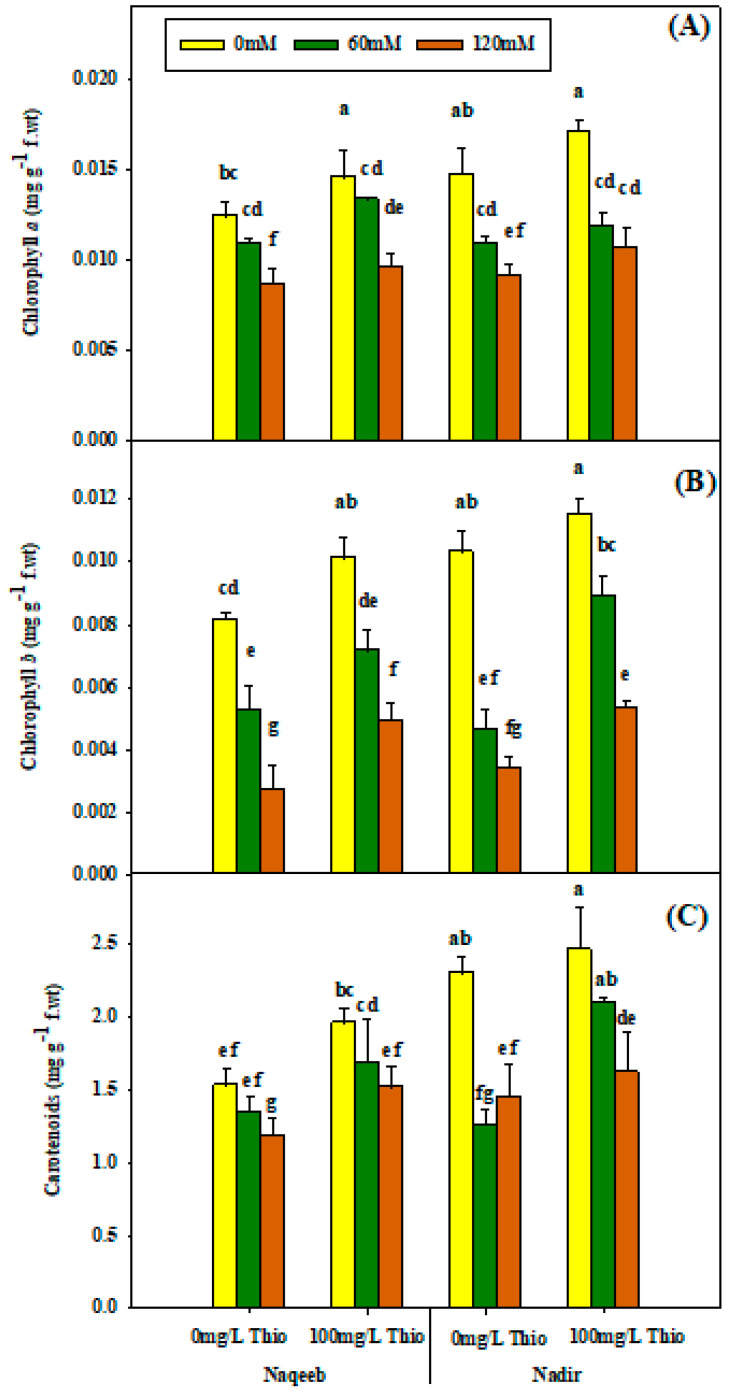
Effects of different foliar applications of thiourea on chlorophyll a (**A**), chlorophyll b (**B**), and carotenoids (**C**) of tomato varieties grown under salinity stress. Error bars = standard error (SE), and bars with different letters indicate significant differences (*p* ≤ 0.05).

**Figure 5:**
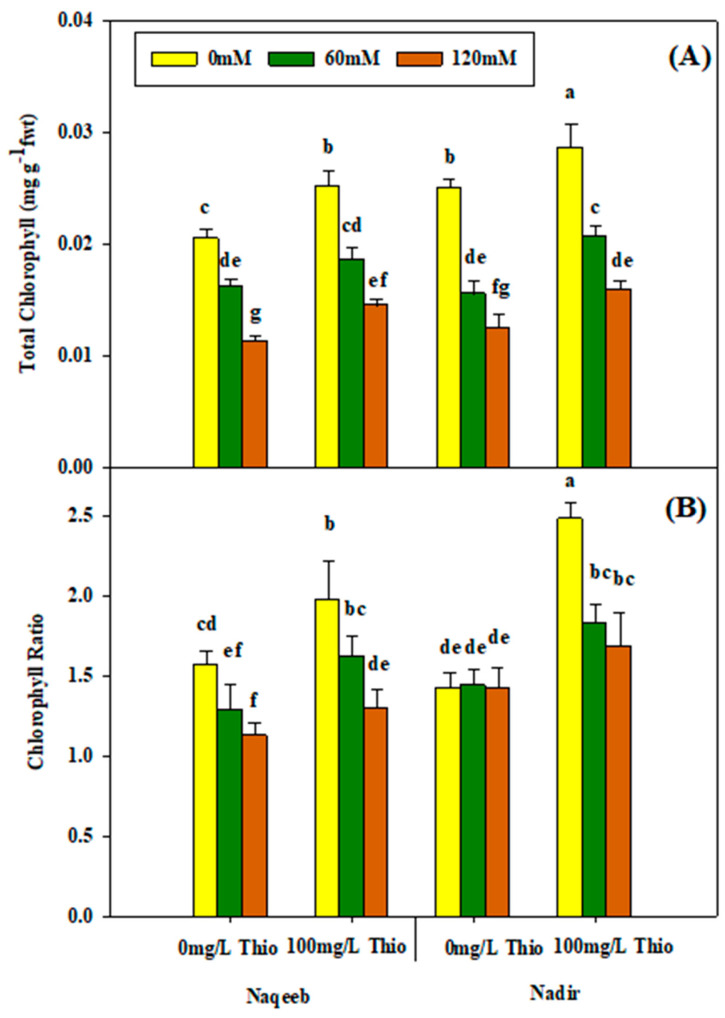
Effects of different foliar applications of thiourea on total chlorophyll (**A**) and chlorophyll a/b ratio (**B**) of two tomato varieties grown under salinity stress. Error bars = standard error (SE), and bars with different letters indicate significant differences (*p* ≤ 0.05).

The analysis of variance showed that chlorophyll *a*, chlorophyll *b*, and carotenoid content were non-significantly affected by salinity stress and thiourea treatment. The application of salt stress (60 mM and 120 mM) caused a non-significant reduction in chlorophyll *a*, chlorophyll *b*, and carotenoid content. The maximum reduction was observed at the 120 mM salt concentration. Thiourea foliar application (100 mg/L) increased chlorophyll *a*, chlorophyll *b*, and carotenoids in both varieties under saline as well as nonsaline conditions. The application of thiourea under nonsaline conditions resulted in the maximum increment in chlorophyll *a* and *b* and carotenoids in the Nadir variety (Table 2 and Figure 4A–C). The same findings were recorded for the chlorophyll ratio and the total chlorophyll. Salinity non-significantly decreased the chlorophyll ratio and the total chlorophyll. The reduction in the chlorophyll ratio and the total chlorophyll was maximum with salt treatment (120 mM) compared to nonsaline conditions. The maximum positive improvement regarding the total chlorophyll and the chlorophyll ratio was observed in the Nadir variety upon the foliar application of thiourea (100 mg/L) under nonsaline and salinity conditions (Table 2 and Figure 5A,B).

### 3.3. Antioxidant Enzymes

**Figure 6:**
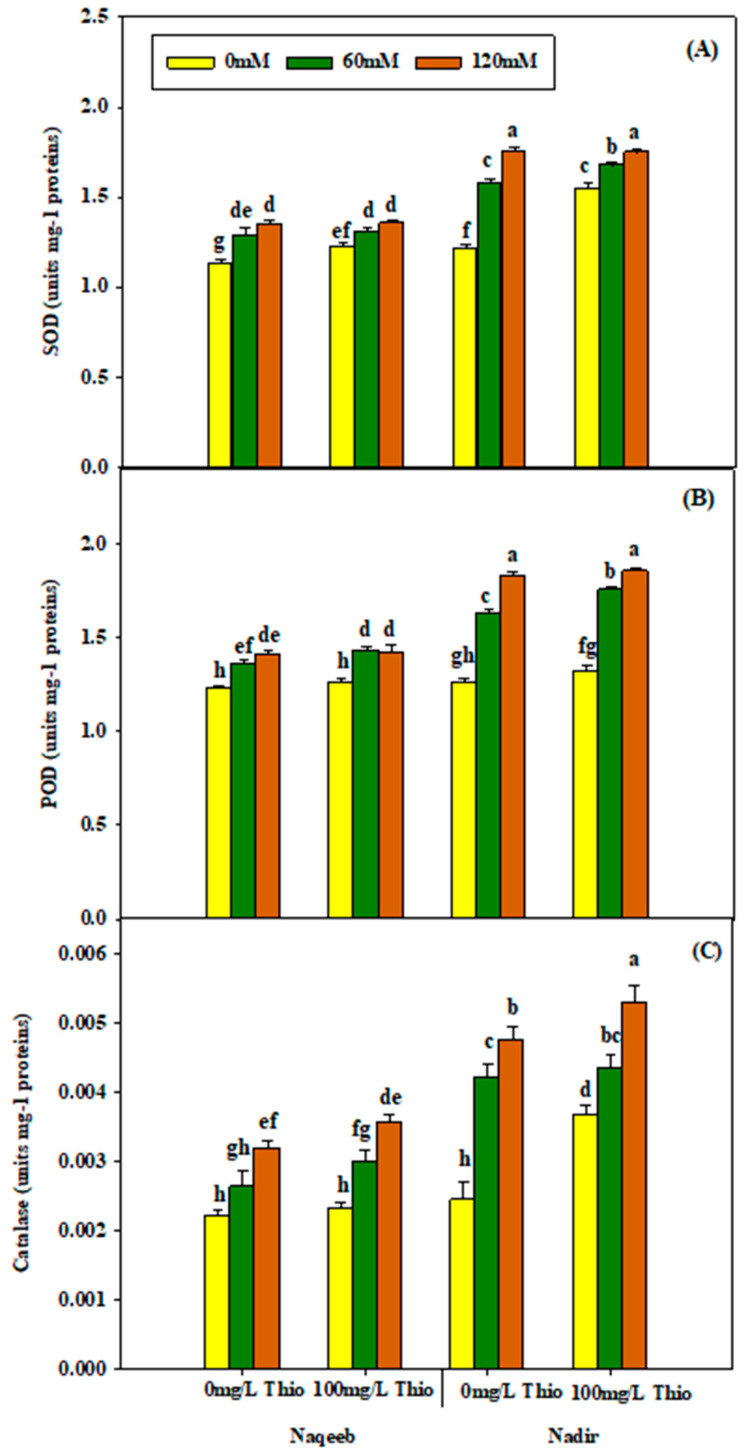
Effects of different foliar applications of thiourea on SOD (**A**), POD (**B**), and CAT (**C**) of tomato varieties grown under salinity stress. Error bars = standard error (SE), and bars with different letters indicate significant differences (*p* ≤ 0.05).

According to the analysis of variance results, salinity significantly affected superoxide dismutase (SOD) and peroxidase (POD), while non-significant results were observed for catalase (CAT). The application of salinity resulted in a significant increase in SOD, peroxidase, and catalase. This increase was maximum under stress treatments (60 mM and 120 mM) as compared to nonsaline conditions. The Naqeeb variety was more affected by salinity than Nadir. ThioU spray (100 mg/L) non-significantly improved POD and CAT content, while significant results were obtained for SOD. The maximum improvement was observed in the Nadir variety under salt stress (120 mM). The foliar application of thiourea improved antioxidant proteins in both varieties under different stress levels (Table 3 and Figure 6A–C).

### 3.4. Biochemical Parameters

**Figure 7:**
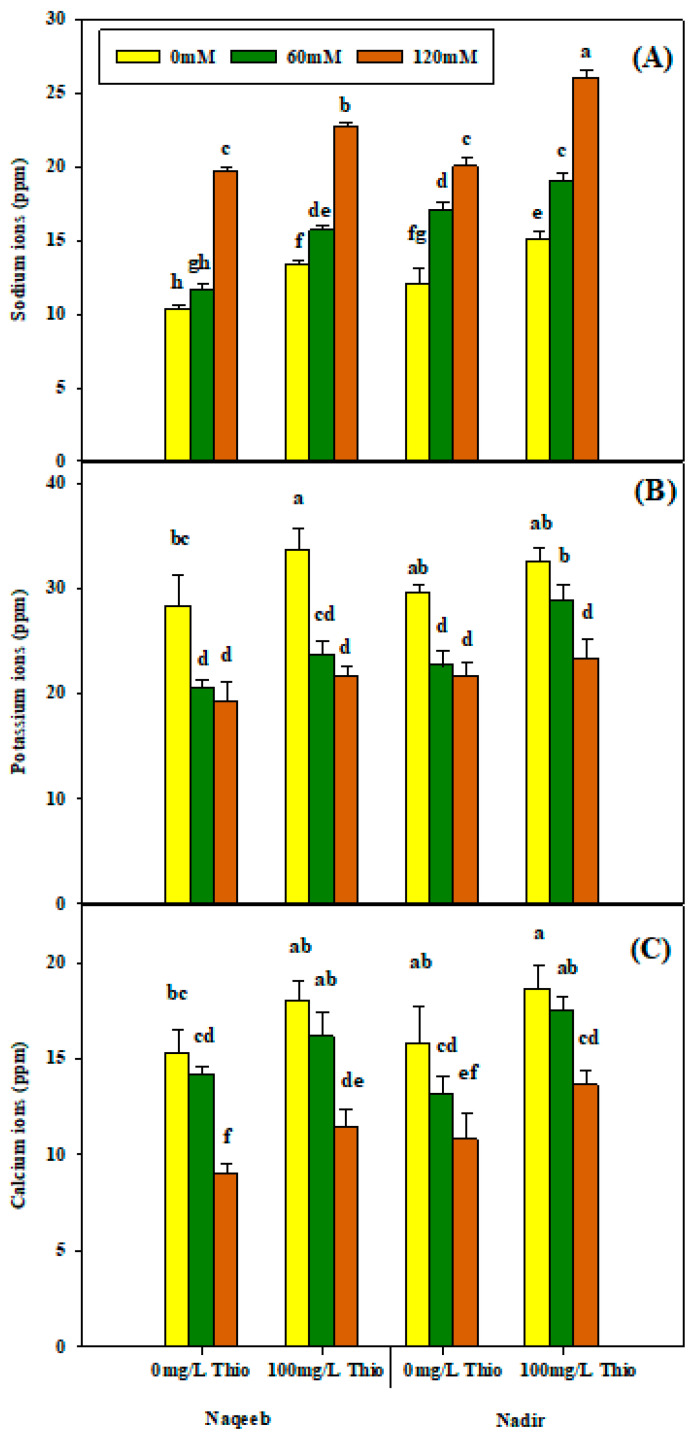
Effects of different foliar applications of thiourea on sodium (**A**), potassium (**B**), and calcium ions (**C**) of tomato varieties grown under salinity stress with foliar application of thiourea. Error bars = standard error (SE), and bars with different letters indicate significant differences (*p* ≤ 0.05).

The analysis of variance results showed that salt non-significantly affected K^+^, Ca^2+^, and Na^+^ ion levels in both varieties. Salinity resulted in a decrease in K^+^ and Ca^2+^ ions, while the accumulation of Na^+^ ions was observed. The maximum reduction in potassium and calcium ions was noted under salt treatments (60 mM and 120 mM) in both varieties. A higher accumulation of sodium ions was detected in Nadir as compared to the Naqeeb variety at the 120 mM salinity level. The foliar application of thiourea (100 mg/L) significantly reduced sodium ions and significantly increased potassium ions, while it did not cause a significant difference in calcium ions in tomato plants grown under each level of salt stress (Figure 7). Thiourea improved the ion concentration in both varieties under different stress levels and mitigated the salt stress effect. The varieties showed differences in response to thiourea application, as more improvement was observed in the Nadir variety as compared to Naqeeb (Table 3 and Figure 7A–C).

### 3.5. Growth and Yield Traits

**Figure 8:**
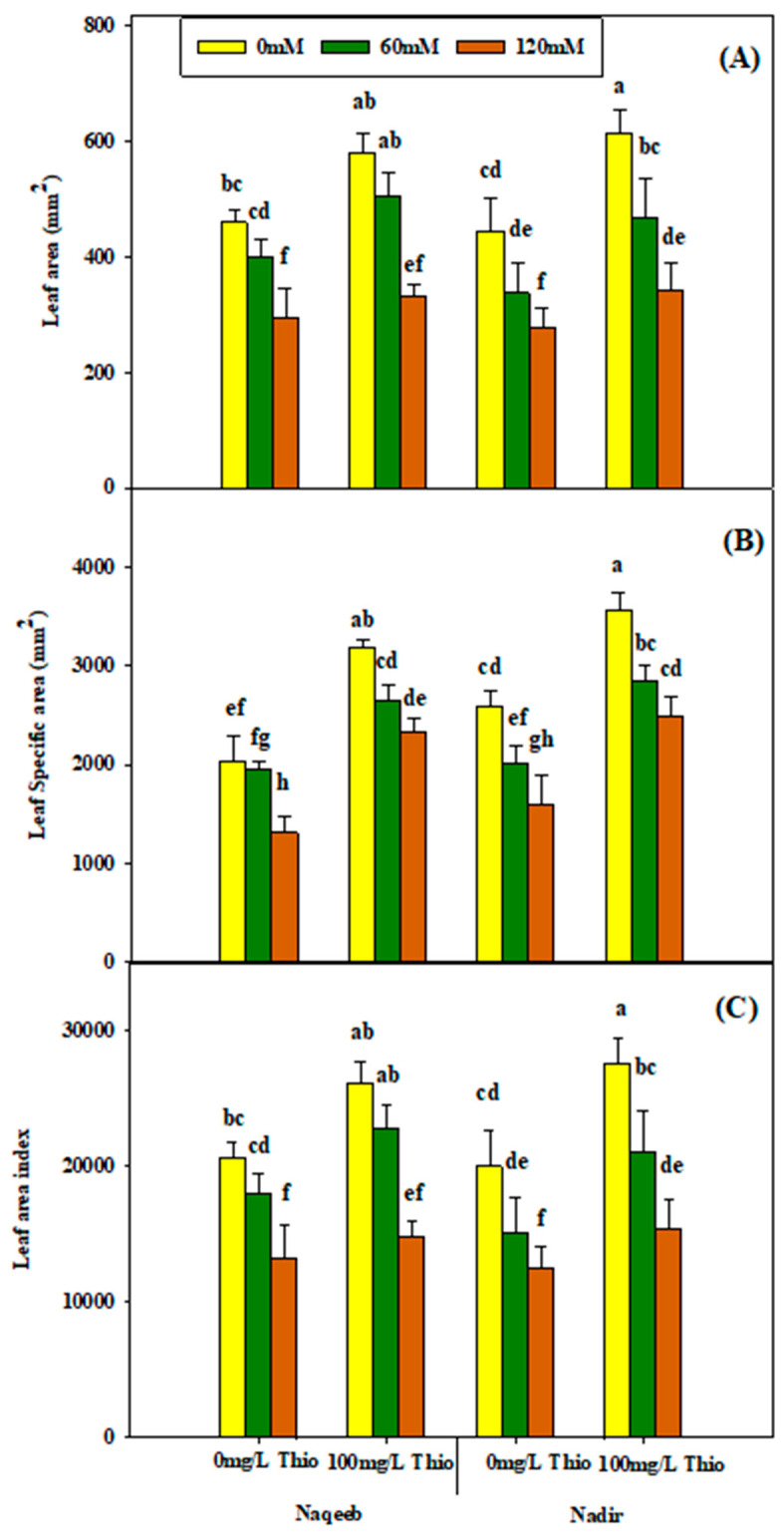
Effects of different foliar applications of thiourea on leaf area (**A**), specific leaf area (**B**), and leaf area index (**C**) of tomato varieties grown under salinity stress with foliar application of thiourea. Error bars = standard error (SE), and bars with different letters indicate significant differences (*p* ≤ 0.05).

Although the interaction effect of salt stress and thiourea was not significant relative to the leaf area, specific leaf area, and leaf area index of tomato plants, their individual effect was significant on the same traits. For instance, salt stress (60 mM and 120 mM) reduced leaf area, specific leaf area, and leaf area index in both varieties, and the maximum reduction was observed at the 120 mM salt level. On the other hand, the foliar application of thiourea resulted in an improvement in the leaf area and leaf area index under salt stress. The maximum leaf area index was observed at the 100 mg/L concentration of thiourea application under nonsaline conditions. However, the Nadir variety showed more improvement in response to thiourea application as compared to Naqeeb under salt stress and nonsaline conditions (Table 4 and Figure 8A–C).

**Figure 9:**
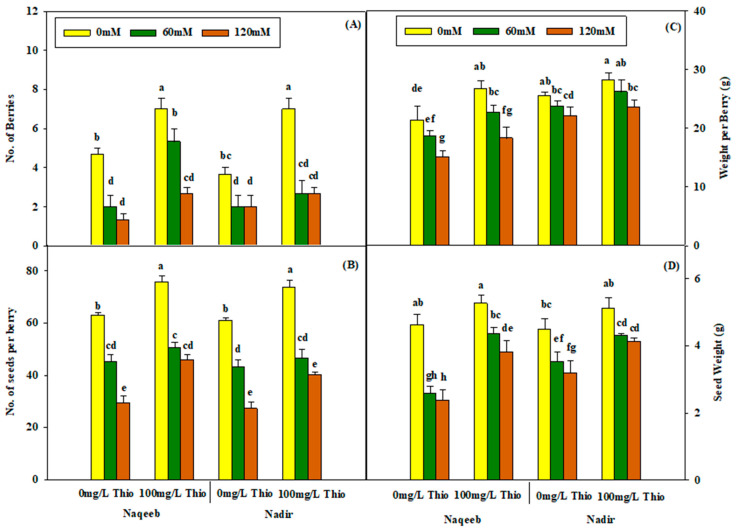
Effects of different foliar applications of thiourea on the number of berries (**A**), number of seeds per berry (**B**), weight per berry (**C**), and seed weight (**D**) of tomato varieties grown under salinity stress. Error bars = standard error (SE), and bars with different letters indicate significant differences (*p* ≤ 0.05).

The number of berries, weight per berry, number of seeds per berry, and seed weight were not significantly different when thiourea (100 mg/L) was applied to tomato plants grown under salt stress at concentrations of 60 mM and/or 120 mM. Salt stress resulted in a reduction in the number of berries, weight per berry, number of seeds per berry, and seed weight in both varieties, while thiourea foliar application increased these yield parameters. However, differences were observed between the two varieties, as the Nadir variety showed maximum yield compared to Naqeeb (Table 4 and Figure 9A–D).

### 3.6. Correlation Analysis

#### 3.6.1. Pearson Correlations

**Figure 10:**
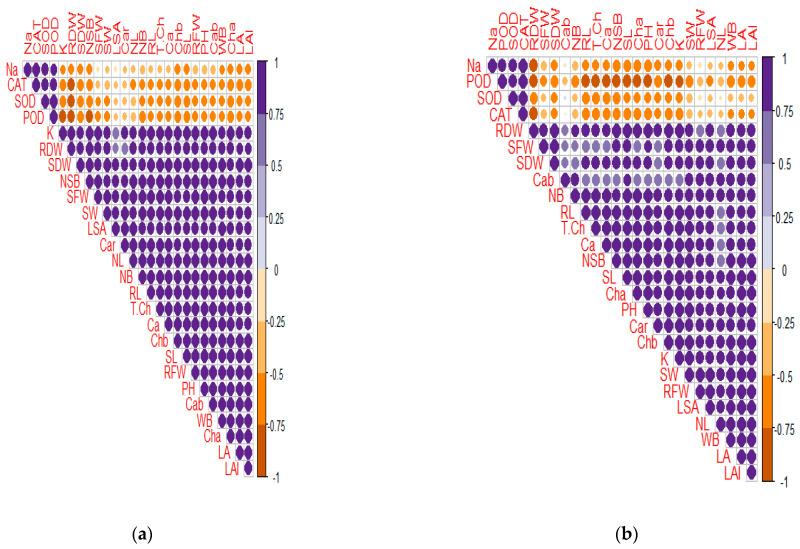
(**a**) Nadir variety; (**b**) Naqeeb variety. Pearson correlations of different morphophysiological and biochemical attributes of tomato (Nadir and Naqeeb). Morphological attributes: shoot dry weight (SDW), root dry weight (RDW), shoot fresh weight (SFW), root fresh weight (RFW), shoot length (SL), root length (RL), plant height (PH), number of leaves (NL), number of berries (NB), number of seed per berry (NSB), seed weight (SW), weight per berry (WB), leaf area (LA), leaf area index (LAI), and specific leaf area (SLA). Physiological attributes: chlorophyll a (Cha), chlorophyll b (Chb), carotenoids (CAR), chlorophyll a and b ratio (Cab), total chlorophyll (T.Ch), superoxide dismutase (SOD), peroxide (POD), and catalase (CAT). Biochemical attributes: sodium (Na^+^), potassium (K^+^), and calcium (Ca^2+^).

The morphological attributes of Nadir and Naqeeb, i.e., SDW, RDW, RF, SF, SL, RL, LA, and PH, strongly positively correlated with ionic contents (K^+^ and Ca^2+^) and photosynthetic pigments (Ch*a*, Ch*b*, CAR, Cab, and T. Ch), while they showed a negative correlation with Na, SOD, POD, and CAT (Figure 10). The morphophysiological attributes of Naqeeb, i.e., RDW, SFW, and Cab, showed a less positive correlation with RL, NB, and CAR. By contrast, other morphological attributes (WB, NSB, NL, SLA, and LAI) were less negatively correlated with Na, SOD, POD, and CAT in Naqeeb as compared to Nadir. Moreover, in Naqeeb, NL, SLA, and Cab did not show any correlation with SOD, POD, CAT, and Na (Figure 10).

#### 3.6.2. Clustered Heatmap

**Figure 11:**
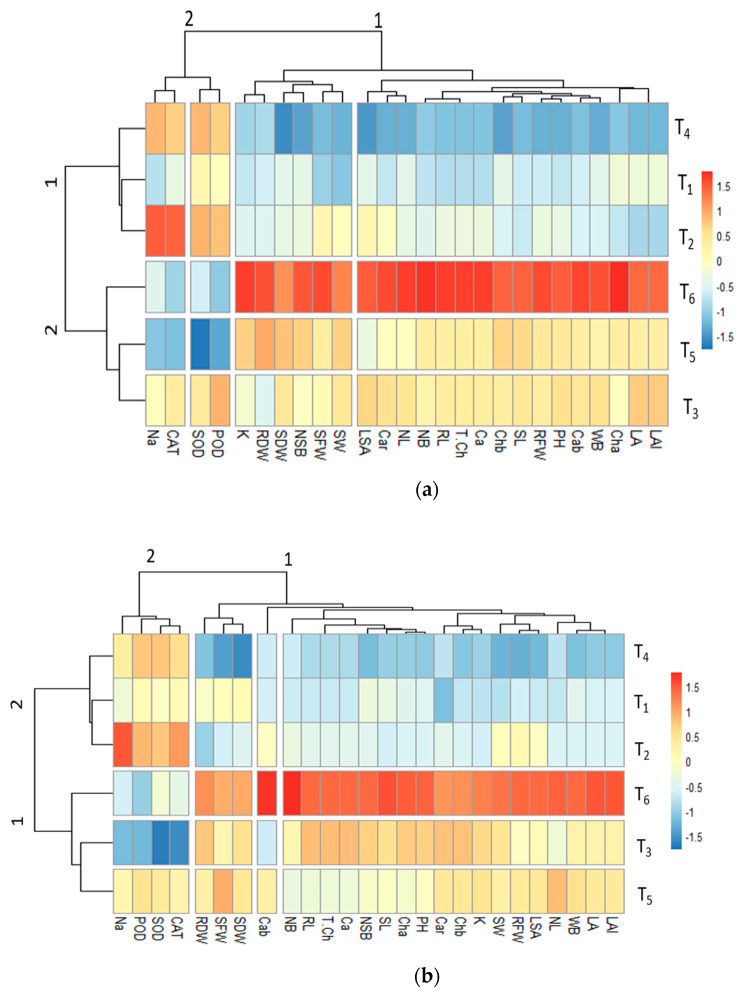
(**a**) Nadir variety; (**b**) Naqeeb variety. Clustered heatmap of different morphophysiological and biochemical attributes of tomato (Nadir and Naqib) under different levels of salt stress and with or without the application of thiourea. Morphological attributes: shoot dry weight (SDW), root dry weight (RDW), shoot fresh weight (SFW), root fresh weight (RFW), shoot length (SL), root length (RL), plant height (PH), number of leaves (NL), number of berries (NB), number of seed per berry (NSB), seed weight (SW), weight per berry (WB), leaf area (LA), leaf area index (LAI), and specific leaf area (SLA). Physiological attributes: chlorophyll a (Cha), chlorophyll b (Chb), carotenoids (CAR), chlorophyll a and b ratio (Cab), total chlorophyll (T.Ch), superoxide dismutase (SOD), peroxide (POD), and catalase (CAT). Biochemical attributes: sodium (Na^+^), potassium (K^+^), and calcium (Ca^+2^). Heatmap represents the separation of treatments into T_1_ (salinity 0 mM + 0 ppm thiourea), T_2_ (salinity 120 mM + 100 ppm thiourea), T_3_ (salinity 60 mM + 0 ppm thiourea), T_4_ (salinity 60 mM + 100 ppm thiourea), T_5_ (salinity 120 mM + 0 ppm thiourea), and T_6_ (salinity 120 mM + 100 ppm thiourea).

The clustered heatmap of Nadir revealed two subclusters, where morphological attributes were grouped with physiological attributes. In cluster 1, the morphological attributes (RFW, SDW, LA, SL, and PH) showed a strong positive association with T_6_ (salinity 120 mM + 100 ppm thiourea). The physiological attributes (Cha, Chb, Na, Ca, and K) of plants treated with T_5_ (salinity 120 mM + 0 ppm thiourea) and T3 (salinity 60 mM + 0 ppm thiourea) showed a less positive association with LA, PL, PH, and PW (Figure 11). At the T_1_ (salinity 0 mM + 0 ppm thiourea) and T_2_ (salinity 120 mM + 100 ppm thiourea) levels, the morphophysiological and biochemical attributes showed less negative associations as compared to the T_4_ level (salinity 60 mM + 100 ppm thiourea), while another cluster showed the grouping of SOD, POD, CAT, and Na, which represented a positive association at the T_2_ (salinity 120 mM + 100 ppm thiourea) and T_4_ (salinity 60 mM + 100 ppm thiourea) levels and a negative association at the T_6_ and T_5_ levels (Figure 11).

The clustered heatmap of Naqib revealed an association of various morphological attributes grouped with physiological attributes. In cluster 1, LAI, LA, WB, NL, RFW, and SW showed a strong positive association at the T_6_ level (salinity 120 mM + 100 ppm thiourea), while at T_2_ (salinity 120 mM + 100 ppm thiourea), RFW and SW did not show any association with other morphological and physiological attributes. In cluster 2, Na was more affected by T_3_ (salinity 60 mM + 0 ppm thiourea) treatment as compared to other treatments, whereas with T_2_ application (salinity 120 mM + 100 ppm thiourea), Na showed a positive association (Figure 11).

## 4. Discussion

Plants face several challenges during their development due to climate change, which has hazardous impacts on plant growth. Salinity stress is considered a major retarding factor in plant growth inhibition [40,41]. Salt stress significantly reduces the growth and yield of tomatoes all around the world. According to [42], the growth of tomato plants was negatively affected by salt stress due to the inhibition of root length (RL) and shoot length (SL). In the present study, the RL and SL of tomato plants decreased by 34% and 32% under salinity conditions, and more decrease was observed in the Naqeeb variety. It is reported that thiourea alleviates harmful salinity impacts and enhances growth in controlled and stressed environments [43]. Thiourea controls several signaling pathways at the germination stage and helps in the alleviation of salinity stress effects. Thiourea helps in enhancing growth under controlled and stressed conditions by balancing cellular redox energetics. These effects might be linked to its ability to break dormancy and stimulate the germination of tomato seeds [44]. The current study revealed that thiourea application improved plant height and root and shoot length under saline stress and control conditions (i.e., the unstressed condition), although the improvements were higher when tomato plants were grown under the unstressed condition. It has been observed that salt stress reduces the FWT and DWT of roots and shoots, which causes a reduction in growth [45]. In the present study, salt stress decreased root and shoot weight by affecting the growth parameters, which were improved by thiourea application at the 120 mg/L concentration.

Photosynthesis is an important function of plant life for fruit development, which is mainly dependent upon the presence of chlorophyll. It has been reported that the concentration of chlorophyll in plants decreases due to salt stress, which can result in low photosynthetic activity. In our present study, salinity at the 120 mM level negatively decreased chlorophyll a and b, and carotenoid concentration in the tomato varieties. In this respect, the growth and development as well as photosynthetic traits of tomato plants were negatively affected when grown under salt stress [46]. However, thiourea can act as an effective growth regulator, which sustains membrane integrity and organelle functions under salt stress [47]. Growth retardation under saline conditions in tomato plants might be due to the substantial decrease in plant photosynthetic ability, as shown by the remarkable reduction in chlorophyll *a* content. The application of thiourea proved beneficial in enhancing the photosynthesis process by improving chlorophyll content in both varieties under saline conditions [48]. In this study, salinity reduced chlorophyll contents, while the foliar spray application of thiourea enhanced the chlorophyll contents by increasing the source-to-sink relationship and osmoregulation in the tomato varieties. Therefore, thiourea can be used in exogenous applications to mitigate abiotic stress (i.e., salt stress).

Tomato plants are very sensitive to stress, and any type of external environmental change like abiotic stress results in the disturbance of the physiological activity of tomato plants, which is vital for their survival [49]. In the present study, salinity reduced the activities of antioxidant enzymes SOD, POD, and CAT in both tomato varieties (Naqeeb and Nadir) due to higher production of ROS. Thiourea serves as a non-physiological ROS scavenger and maintains a high-antioxidant defense system by modulating the metabolism and signal transduction pathways in tomato plants [50]. The application of thiourea at 120 mg/L greatly enhanced these enzymes and improved antioxidant activities to scavenge ROS produced under salt stress. It was also observed that thiourea application (500 ppm and 1000 ppm) improved antioxidant enzyme activities by reducing sodium ion concentration and salinity stress [51] and also prevented protein degradation. Thiourea foliar spray application in tomato plants inhibited protein loss and protease activity and increased CAT and POD activities in wheat under oxidative stress [52].

Ion homeostasis is an important mechanism in plants, but in previous studies, it has been recorded that, under salt stress, ionic balance is strongly disturbed, which affects plant growth by inhibiting the uptake of essential nutrients like potassium and calcium [29,53,54]. Plants experience osmotic and ionic stresses under saline conditions. Initial indicators include a high number of sodium and intracellular calcium ions, along with ROS accumulation [55]. High sodium leads to an increase in sodium pumping outside the cytoplasm and sequestration inside the vacuole. This leads to the efflux of potassium from vesicles to maintain homeostasis. Thiourea helps to promote potassium uptake in tomato plants under saline conditions [52]. This study’s findings revealed that salinity caused an increment in sodium ions, which can block the uptake of soil nutrients. This resulted in a decrease in the concentration of potassium and calcium ions in the roots. In the Naqeeb variety, sodium ions were absorbed in higher concentrations, which negatively affected growth. In this study, thiourea application demonstrated a positive effect in lowering sodium ion concentration and enhancing potassium and calcium uptake from roots. An increase in potassium ions resulted in the mitigation of salt stress.

Salinity negatively affected the number of berries, number of seeds, and their weight in Nadir and Naqeeb, while the maximum reduction was observed in Nadir as compared to Naqeeb. Salt stress causes a reduction in the yield of crop plants due to disruption in photosynthesis, low uptake of mineral ions, higher accumulation of sodium and chloride ions, and poor growth [56,57]. Thiourea application enhances the growth and photosynthetic rate of plants by increasing photosynthetic pigments [58]. It has also been observed that thiourea application increases photosynthetic activity in sesame under salinity conditions and ultimately enhances yield [59]. On the other hand, the grain yield of wheat plants grown with a combination of thiourea and bagasse ash application was 9.27 t ha^−1^ and significantly increased by 9.60, 14.67, and 29.27% in comparison to the foliar application of thiourea, the soil application of bagasse ash, and control treatments, respectively [28]. In the present study, thiourea enhanced the number of berries, number of seeds, and seed weight in both varieties. According to Hussien Ibrahim et al. [60], the leaf area of plants is reduced under salinity stress. The findings of the present study showed that the leaf surface area was deceased by salinity stress. Thiourea increased the surface area of both varieties, leading to a higher exchange of CO_2_ between the leaves and the environment and an increase in the photosynthetic activities of Naqeeb and Nadir under salt stressed and controlled conditions.

## 5. Conclusions

Salinity significantly reduced the growth and productivity traits of two varieties of tomato plants. For example, salt stress reduced tomato the plant height by 20%, fresh shoot weight by 50%, dry shoot weight by 78%, fresh root weight by 43%, dry root weight by 84%, root length by 34%, shoot length by 32%, shoot K^+^ by 47%, Ca^2+^ by 70%, chlorophyll a by 30%, chlorophyll b by 67%, and the number of seeds per berry by 53%, while shoot Na^+^ ions were increased by 90% in comparison to those grown with the control treatment. However, nutrient applications including thiourea (i.e., 100 mg/L) could mitigate environmental stresses such as salinity and enhance growth and yield by improving the morphological, physiological, and yield traits of Naqeeb and Nadir tomato varieties. For example, the exogenous application of thiourea (i.e., 100 mg/L) significantly enhanced dry root weight by 25% and the number of seeds per berry by 20% in comparison to untreated plants with thiourea when grown under salt stress. The current study demonstrates that thiourea application positively affected plant defense against salt stress in the Naqeeb and Nadir tomato varieties. Further investigation is required to determine the effect of thiourea treatment in combination with other nutrients to mitigate salt stress in tomato plants.

## Figures and Tables

**Table 1:** Analysis of variance results of shoot and root traits of tomato grown under salinity stress with the foliar application of thiourea.

SOV	SL	SFW	SDW	RL	RFW	RDW	PH
ThioU	36.20 **	7.12 ***	0.59 ***	0.85 ***	0.26 ***	0.27 *	49.93 ***
Sal	103.11 ***	3.27 ***	1.34 ***	1.60 ***	0.003 ns	3.19 ***	100.26 ***
Var	2.00 ns	4.85 ***	1.02 ***	0.58 ***	0.15 ***	0.57 ***	2.77 ns
ThioU * Sal	1.73 ns	1.03 **	0.05 ***	0.01 ns	0.001 ns	0.03 ns	0.33 ns
ThioU * Var	0.006 ns	0.87 *	0.07 ***	0.10 ns	0.003 ns	6.9444 × 10^−5^ ns	0.44 ns
Sal * Var	3.22 ns	0.004 ns	0.11 ***	0.02 ns	0.004 ns	0.38 ***	8.39 ns
ThioU * Sal * Var	1.68 ns	0.01 ns	0.01 ns	0.005 ns	6.1944 × 10^−4^ ns	0.007 ns	0.46 ns
Error	3.89 <-	0.13 <-	0.004 <-	0.04 <-	0.003 <-	0.03 <-	3.39 <-

* Significant at *p* ≤ 0.05, ** Significant at *p* ≤ 0.01. *** Significant at *p* ≤ 0.001, ns = non-significant, SOV = sum of variance, SL = shoot length, SFW = shoot fresh weight, SDW = shoot dry weight, RL = root length, RFW = root fresh weight, RDW = root dry weight, PH = plant height.

**Table 2:** Analysis of variance results regarding the physiological traits of tomato plants grown under salinity stress with foliar application of thiourea.

SOV	Chl. *a*	Chl. *b*	Car	Chl. *a*/*b* Ratio	Total Chl.
ThioU	2.68 × 10^−5^ **	4.53 × 10^−5^ ***	1.30 ***	1.71 ***	3.68 × 10^−5^ **
Sal	9.85 × 10^−5^ ***	1.06 × 10^−4^ ***	1.25 ***	0.71 ***	3.84 × 10^−4^ ***
Var	6.27 × 10^−6^ ns	8.38 × 10^−6^ **	0.97 ***	0.48 **	1.26 × 10^−4^ ***
ThioU * Sal	4.96 × 10^−6^ ns	1.73 × 10^−6^ ns	0.10 ns	0.20 *	8.78 × 10^−6^ ns
ThioU * Var	8.27 × 10^−8^ ns	4.02 × 10^−7^ ns	9.58 × 10^−4^ ns	0.15 ns	1.02 × 10^−6^ ns
Sal * Var	1.33 × 10^−6^ ns	1.49 × 10^−6^ ns	0.21 ns	0.02 ns	5.18 × 10^−7^ ns
ThioU * Sal * Var	1.006 × 10^−6^ ns	2.17 × 10^−6^ ns	0.13 ns	0.08 ns	2.64 × 10^−6^ ns
Error	2.29 × 10^−6^ <-	1.02 × 10^−6^ <-	0.06 <-	0.05 <-	3.42 × 10^−6^ <-

* Significant at *p* ≤ 0.05, ** Significant at *p* ≤ 0.01. *** Significant at *p* ≤ 0.001, ns = non-significant, SOV = sum of variance, Chl. *a* = chlorophyll *a*, Chl. *b* = chlorophyll *b*, Car = carotenoids, Total Chl. = total chlorophyll.

**Table 3:** Analysis of variance results regarding the biochemical traits of tomatoes grown under salinity stress with the foliar application of thiourea.

SOV	SOD	POD	CAT	Na^+^	K^+^	Ca^2+^
ThioU	0.85 ***	0.58 ***	1.51 × 10^−5^ ***	61.36 ***	116.64 ***	73.67 ***
Sal	0.23 ***	0.42 ***	7.12 × 10^−6^ ***	275.52 ***	293.32 ***	103.0 ***
Var	0.07 ***	0.02 ***	1.91 × 10^−6^ ***	110.25 ***	32.49 ns	7.56 ns
ThioU * Sal	0.02 ***	0.10 ***	6.08 × 10^−7^ **	6.69 **	6.19 ns	0.21 ns
ThioU * Var	0.02 ***	0.002 ns	2.66 × 10^−7^ ns	0.25 ns	0.001 ns	2.00 ns
Sal * Var	0.03 ***	0.005 *	1.34 × 10^−7^ ns	2.25 ns	10.12 ns	2.77 ns
ThioU * Sal * Var	0.01 **	1.30 × 10^−4^ ns	3.68 × 10^−7^	4.75 *	5.55 ns	1.09 ns
Error	0.001 <-	0.001 <-	8.24 × 10^−8^ <-	0.91 <-	7.77 <-	3.51 <-

* Significant at *p* ≤ 0.05, ** Significant at *p* ≤ 0.01. *** Significant at *p* ≤ 0.001, ns = non-significant, SOV = sum of variance, SOD = superoxide dismutase, POD = peroxidase, CAT = catalase, Na^+^ = sodium ions, K^+^ = potassium ions, Ca^2+^ = calcium ions.

**Table 4:** Analysis of variance results for the leaf and yield traits of tomato grown under salinity stress with the foliar application of thiourea.

SOV	NOL	LA	SLA	LAI	NOB	WB	NOS	Seed Weight
ThioU	1122.25 ***	97,552.1 ***	661,639.31 **	197,543,025 ***	34.02 ***	178.00 ***	78.02 *	0.73 ns
Sal	568.36 ***	136,048.69 ***	2,462,494.3 ***	2.75499 × 10^8^ ***	46.58 ***	96.48 ***	3307.86 ***	7.42 ***
Var	600.25 ***	1906.77 ns	7,766,442.1 ***	3,861,225 ns	2.25 ns	92.89 ***	1013.36 ***	9.62 ***
ThioU * Sal	91.08 *	7035.19 ns	92,004.92 ns	14,246,269 ns	4.52 **	8.44 ns	2.52 ns	0.44 ns
ThioU * Var	8.02 ns	2567.11 ns	6825.7233 ns	5,198,400 ns	1.36 ns	8.45 ns	8.02 ns	0.56 ns
Sal * Var	1.58 ns	2923.02 ns	61,653.69 ns	5,919,131.3 ns	1.58 ns	2.17 ns	92.19 **	0.37 ns
ThioU * Sal * Var	38.86 ns	100.86 ns	21,788.93 ns	204,243.75 ns	0.52 ns	0.21 ns	2.52 ns	0.18 ns
Error	18.11 <-	5511.80 <-	84,203.69 <-	11,161,406 <-	0.77 <-	6.53 <-	14.41 <-	0.21 <-

* Significant at *p* ≤ 0.05, ** Significant at *p* ≤ 0.01. *** Significant at *p* ≤ 0.001, ns = non-significant, SOV = sum of variance, NOL = number of leaves, LA = leaf area, SLA = specific leaf area, LAI = leaf area index, NOB = number of berries, WB = eight per berry, NOS = number of seeds.

## Data Availability

Data will be provided if required.
